# Allowing time for 8+ hours of sleep: identification and validation of important beliefs using the reasoned action approach

**DOI:** 10.3389/fpsyg.2024.1402322

**Published:** 2024-08-07

**Authors:** Michael J. Tagler

**Affiliations:** Ball State University, Muncie, IN, United States

**Keywords:** sleep duration, elicitation study, reasoned action approach, theory of planned behavior, sleep beliefs

## Abstract

**Introduction:**

The present studies advance research using the Reasoned Action Approach to understand sleep behavior. Identification of the modal salient beliefs that individuals hold regarding their sleep habits is necessary to understand the attitudes, perceived normative pressure, and perceived behavioral control (PBC) that individuals hold.

**Methods:**

Belief elicitation (Study 1) and follow-up validation (Study 2) studies of undergraduates at a Midwestern USA university were conducted to identify readily accessible and important beliefs regarding allowing time for 8+ hours of sleep each night.

**Results:**

Important attitude relevant beliefs included positive effects on mood, thinking, health, and productivity. Important normative beliefs were perceived injunctive pressure from family, health professionals, and significant others. Because of the strong influence of PBC on intentions/behavior, most important were control beliefs about the need for good time management.

**Discussion:**

The present studies increased our understanding why many individuals do not allow time to obtain adequate sleep. Identification of the beliefs that distinguish between those who intend to allow time for adequate sleep and those who do not is a necessary step toward the design of effective interventions to improve sleep duration. The results indicate that a focus on increasing time management skills and PBC may be an effective approach for sleep interventions.

## 1 Introduction

Sleep is a biological requirement, but many people fall short of consistently getting enough for optimal health and well-being. Recent research has applied the Reasoned Action Approach (RAA; Fishbein and Ajzen, 2010) to identify the factors that contribute to healthy sleep behavior. The present paper reports the results of two studies designed to advance this work by identifying important behavioral, normative, and control beliefs regarding allowing enough time to sleep for 8+ hours per night. Identification of these beliefs is critical toward the development of effective interventions to improve sleep.

Empirically based, expert consensus is that adults need 7–9 h of sleep for optimal health (Hirshkowitz et al., 2015; Watson et al., 2015). But how many adults meet this need? Studies that employ single item self-report measures of sleep duration find approximately 1/3rd of adults report less than 7 h per night on a typical night (Ford et al., 2015; Liu et al., 2016; Centers for Disease Control and Prevention (CDC), 2020). Studies that measure sleep duration more thoroughly over time (e.g., sleep diaries, actigraphy) provide even more cause for concern. For example, Scott et al. (2024) conducted a powerful analysis of sleep duration from over 67,000 adults using a mattress sleep sensor over 9-months. Only 15% of these adults slept between 7 and 9 h for at least five nights per week, and only 2% slept 7–9 h for six nights per week. Obtaining sufficient sleep on a consistent nightly basis is a challenge for many, if not most adults.

The prevalence of insufficient sleep is concerning because study after study demonstrate negative outcomes. A thorough review is well beyond the purpose of this article, requiring contributions from multiple fields (e.g., Altevogt and Colten, 2006). The outcomes include negative effects on myriad aspects of physical health, psychological well-being, social behavior, and society. For example, insufficient sleep is a risk factor for obesity (Chaput et al., 2023), diabetes (Gottlieb et al., 2005), cardiovascular disease (Ayas et al., 2003), and all-cause mortality (Grandner et al., 2010). Short sleep is associated with depression (Dong et al., 2022), anxiety (Koffel and Watson, 2009), and has a negative impact on mood states (Short et al., 2020). Moreover, sleep duration is related to self-control (Guarana et al., 2021), aggression (Van Veen et al., 2022), and the quantity/quality of social relationships (Gordon et al., 2021). Sleep deprivation impairs executive functioning, including attention, learning, memory, and reasoning (Walker, 2008; Lim and Dinges, 2010; Chambers and Payne, 2015). These impairments are not just personal, as they lead to public, occupational, and safety threats including impaired (drowsy) driving (Bioulac et al., 2017).

## 2 The reasoned action approach to predicting and changing behavior

The Reasoned Action Approach (RAA; Fishbein and Ajzen, 2010) is designed to identify the social-cognitive factors that distinguish between people who do or do not engage in a specific behavior. The RAA developed from prior iterations of the model that included the Theory of Reasoned Action (Ajzen and Fishbein, 1980) and the Theory of Planned Behavior (TPB; Ajzen, 1985). In this approach, behavioral intention, “a person’s readiness to perform a behavior” (Fishbein and Ajzen, 2010, p. 39), is the most proximal and strongest predictor of engaging in the behavior. Intentions are measured by asking the individual to estimate the likelihood that they expect to perform the behavior, within a specified time and/or context. Furthermore, the RAA specifies three direct predictors of intention: attitude, perceived normative pressure (PNP), and perceived behavioral control (PBC).

Attitude, PNP, and PBC are the result of specific social and cognitive beliefs (Fishbein and Ajzen, 2010). Attitude is the overall evaluation that results from the collection of beliefs one holds regarding the expected outcomes of personally engaging in the behavior. For example, a positive attitude toward allowing time for at least 8 h of sleep should logically result from holding beliefs that doing so will feel enjoyable (experiential belief) and will result in improved health (instrumental belief). PNP results from beliefs regarding the expectations and influence of others. For example, high PNP should result from believing that important others such as family want the individual to engage in the behavior (injunctive norm), and the belief that important others engage in the behavior themselves (descriptive norm). Lastly, PBC represents beliefs about ability and self-sufficiency to carry out the behavior. For example, high PBC should reasonably result from confidence in one’s capability to allow time for at least 8 h of sleep nightly (capacity belief) and the belief that they can perform the behavior if they choose to (autonomy belief).

A large body of research supports Fishbein and Ajzen (2010) approach, particularly in health domains (e.g., Albarracın et al., 2001; Downs and Hausenblas, 2005; Collins and Carey, 2007). A recent meta-analysis (McEachan et al., 2016) found that the RAA accounts for an average of 59% of the variance in health behavior intentions, and 31% of the variance in behavior.

## 3 Reasoned action-based sleep research

The results of studies applying reasoned action theories to sleep behavior are promising (see Mead and Irish, 2020 for a recent review). These studies find the model to predict both behavioral intention and engagement (generally self-reported) in a variety of sleep behaviors. Kor and Mullan (2011) used the TPB framework to examine a composite of three sleep hygiene behaviors (making the bedroom restful, avoiding going to sleep thirsty or hungry, avoiding anxiety/stress prior to bedtime). Perceived norms and PBC were significant predictors of intention, and intention and PBC were both predictive of self-reported behavior 1 week later. PBC was the strongest predictor of both intention and behavior. Strong et al. (2018) reported a results with Iranian adolescents.

Rather than sleep hygiene, some studies focused on sleep duration. Knowlden et al. (2012) found the TPB to significantly predict intentions and self-reported “sleeping 7–8 h per night.” Notably, this study found PBC to be the strongest predictor. Similar results were reported by Sheeran et al. (2002) and Lao et al. (2016). Branscum et al. (2020) used the RAA to predict “getting 7–9 h of sleep each night,” comparing college students who reported they do not get 7–9 h (behavioral adoption group) to students who reported they were do (behavioral maintenance group). The groups differed in important ways (e.g., intention and PBC were much lower in the adoption group). But, similar to Knowlden et al., PBC was the strongest predictor of intention for both groups (especially the capacity component).

Across four studies with college students, Tagler et al. (2017) provided further support that the RAA predicts intentions to engage in both sleep hygiene behaviors and sleep duration. The Tagler et al. work is notable in two respects. First, sleep hygiene behaviors were examined as a category and separately compared. In studies 1a and 1b, sleep hygiene was defined to participants as including all the following: Allowing for at least 8 h of sleep per night, keeping a regular sleep schedule, exercising regularly but not close to bedtime, and avoiding/limiting each of the following close to bedtime: caffeine, alcohol, and large meals. This approach treats sleep hygiene as a behavioral category (Fishbein and Ajzen, 2010). All RAA predictors were statistically significant and combined to account for a very large proportion (70%) of variability in intentions, with PBC as the strongest predictor. In Study 2, the six sleep hygiene behaviors were examined individually to separate samples of participants. The RAA predicted intentions to engage in each behavior (accounting for 66–75% variability), but differences emerged. Attitude was the strongest predictor of intention for all behaviors, with one notable exception: PBC was the strongest predictor of intention to allow for at least 8 h of sleep. Mean PBC was significantly lower for allowing for 8 h than it was for the other behaviors. Moreover, intention to allow for 8 h of sleep was significantly lower than intentions to engage in the other behaviors, save for avoiding caffeine.

The second important contribution from Tagler et al. (2017) is the use of actigraphy in their Study 3. Whereas previous published studies relied exclusively on self-reported recollections of behavioral engagement, Tagler et al. used wrist actigraphy (activity monitors) to record sleep duration over 7-days. Although all predictors were significant, PBC was again the strongest predictor of intention to “allow time for at least 8 h of uninterrupted sleep over the next 7 days.” However, PBC did not add to the prediction of actual sleep duration. Rather, intention was the only significant predictor of sleep duration (both actigraphy and sleep diary self-reports).

Mead and Irish (2022) also used actigraphy in an RAA study on “sleep opportunity.” They operationalized “sleep opportunity” as the number of minutes between the time to bed and out of bed (recorded by the participant pressing a marker button on the actiwatch). Moreover, Mead and Irish used ecological momentary assessment (EMA) in which participants reported their attitudes, PNP, PBC, and intention four times per day. This powerful repeated-measures design revealed that PBC was the only significant predictor of future (later that same day) intentions. Also, PBC combined with intention to predict sleep opportunity.

## 4 Moving from predicting to understanding and improving sleep

Thus, studies show the RAA constructs of attitude, PNP, and PBC are useful to predict sleep intentions and behaviors. Often, PBC is a particularly important predictor. To continue this work toward the design of interventions to improve sleep, research is needed to identify the most important beliefs that underlie attitudes, PNP, and PBC. In the terminology of Fishbein and Ajzen (2010), this task is accomplished with belief elicitation studies in which small samples of participants respond to open-ended questions to identify specific behavioral, normative, and control beliefs regarding engaging in the behavior. The beliefs that participants most often mention are considered the set of “modal salient beliefs” and “readily accessible.” Next, an expectancy-value model is used to determine which of the salient beliefs are most important (i.e., the best predictors of overall attitude, PNP, and PBC). Of these, the beliefs that have room to improve and are most amenable to influence can be targeted in an intervention (Ajzen and Schmidt, 2020).

To date, only two sleep behavior belief elicitation studies have been published. Robbins and Niederdeppe (2015) solicited college student beliefs toward “sleeping for between 8 and 9 h at night, most nights per week.” Beliefs involving stress and time (e.g., “having less stress,” “manage time effectively,” “not enough time to do work”) were the strongest predictors of intention, and of self-reported sleep duration. From these results, the authors suggest the design of stress and time management interventions. However, because they directly correlated beliefs with intention/behavior, results reported by Robbins and Niederdeppe (2015) may not fully describe the way in which beliefs lead to intention/behavior. In the RAA, the effect of beliefs on intentions and behavior is indirect (i.e., mediated by attitude, PNP, and PBC). As such, beliefs are validated by examining their correlation with direct measures of attitude, PNP, or PBC. An additional limitation in the Robbin and Niederdeppe study was that the direct measures of attitude, PNP, and PBC were very short (three questions each). This concern most evident on perceived norms, on which all three questions assessed injunctive pressure (descriptive pressure was omitted) and responses did not combine to form a reliable scale. Lastly, it can be argued that “sleeping for 8–9 h” is a goal, not a behavior (Fishbein and Ajzen, 2010). The issue here is that behavioral engagement does not always result in goal attainment (e.g., dieting may not result in weight loss). In the case of *obtaining* 8–9 h of sleep, even when one engages in good sleep habits (e.g., going to bed at a consistent time) there are factors that may interfere (e.g., bad dreams). The RAA best predicts specific volitional behaviors (e.g., avoiding caffeine), rather than desired goals (e.g., uninterrupted 8 h of sleep). Moreover, interventions results are more meaningful when the focus is on behavior change rather than goals.

Vézina-Im et al. (2023) conducted an elicitation study comparing the beliefs of adults toward sleep behaviors that included “avoiding screen use in bed” and “having a regular sleep schedule.” Although the focus of the study was to compare adults with and without diabetes, both groups shared many of the same beliefs. For example, common attitude-relevant beliefs were that reducing screen time results in feeling more relaxed, easier to fall asleep, and better sleep quality. Moreover, a salient belief was that a regular sleep schedule results in more energy and better mood. Regarding normative beliefs, significant others, friends, and family were reported as influential. Control beliefs included that avoiding screen time is facilitated by silencing electronic devices and by reading a book. On keeping a regular sleep schedule, most often cited barriers were social activities and work/studies. With the emphasis on specific behaviors, the Vézina-Im et al. study provides a good template for how to conduct belief elicitation studies on sleep habits.

In a follow-up report by the same researchers (Vézina-Im et al., 2024), PBC (but not intention) was shown to predict self-reported screen use and regular sleep schedule. Furthermore, the authors sought to identify the most important control beliefs. On electronic device use, a significant predictor of behavior was degree of agreement with “it would be easier if I removed alerts on my cell phone.” On having a regular sleep schedule, “I feel capable…even if I have many things to do” was a significant predictor. Because they directly correlated beliefs with behavior, similar to the approach used by Robbins and Niederdeppe (2015), the results here also do not fully describe the way in which beliefs lead to behavior. Moreover, both Robbins and Niederdeppe and Vézina-Im et al. did not apply the expectancy value measurement approach described by Fishbein and Ajzen (2010), in which separate questions of belief strength and evaluation/motivation/power are separately measured.

Like belief elicitation studies, RAA-based sleep interventions are rare. Lin et al. (2018) designed an intervention to improve the sleep hygiene of Iranian adolescents partially based on RAA constructs. Over a 2-month period, adolescents attended four 1-h group sessions emphasizing avoiding going to bed hungry/thirsty, reducing stress provoking activities before bedtime, and making the bedroom restful. The authors designed the session content to logically correspond to the RAA predictors. For example, to improve attitudes the adolescents were presented with information on the importance of sleep for health and daily functioning. Normative pressure was targeted by having parents attend one of the sessions and provide feedback on their adolescent’s sleep habits. To increase PBC, recommendations were provided on improving the sleep environment. Furthermore, participants created plans for carrying out the behaviors. At 1-month and 6-month follow-up, significant improvements were reported on measures of attitude, PNP, PBC, intention, self-reported sleep behavior, and sleep duration. The behavior changes were mediated by changes in PNP and PBC (but not by attitude). The Lin et al. results represent an important contribution to the design of RAA interventions. However, a limitation is that it was designed at a conceptual level rather than empirical (i.e., no belief elicitation study was conducted to inform the intervention on which beliefs to target).

Zhao et al. (2019) tested an intervention to “reduce late evening bedtime electronic device use” among young adults. Upon finding that attitude and PBC were significant predictors of intention, the authors created an intervention consisting of an educational message (e.g., “using devices can disrupt sleep,” “you can control device usage”). At 1 week follow-up, results included improved intentions and a reduction in self-reported device use. Unfortunately, post-intervention attitude, PNP, and PBC measures were not taken and thus it is unknown if changes in these constructs mediated the results. Like the Lin et al. (2018) intervention, Zhao et al. designed their intervention at a conceptual level based on TPB constructs rather than from the results of an empirical, belief-elicitation study.

## 5 Rationale and overview of the present studies

Research indicates that the RAA is effective for predicting sleep behaviors. To advance this work, belief elicitation studies are needed to identify the modal salient beliefs that individuals hold regarding engaging in sleep behaviors. Further, research is needed to determine which of these salient beliefs are the most important (strongest) predictors of attitude, PNP, and PBC. Doing so will increase understanding of the beliefs that distinguish between those who intend to engage in the behaviors and those who do not. Moreover, it is critical to identify beliefs that are most amenable to change, and thus the most promising targets for intervention.

Two studies were conducted toward these goals. Study 1 was a belief elicitation study to identify the modal salient behavioral, normative, and control beliefs regarding “allowing time for adequate sleep duration (at least 8 h of sleep each night).” Study 2 was a validation study to determine which of the identified beliefs most strongly predict attitudes, PNP, and PBC.

## 6 Study 1 method

### 6.1 Participants

Undergraduates (*N* = 138) at a large Midwestern USA university were recruited from an introductory psychology participant pool and received course credit for their participation. Their mean age was 19.09 years (SD = 4.84). Participants reported as White/Caucasian (*n* = 87, 63.04%), Black/African American (*n* = 20, 14.49%), Hispanic (*n* = 6, 4.34%), Asian/Pacific Islander (*n* = 2, 1.44%), other/prefer to not say (*n* = 8, 5.80%), and 15 (10.87%) did not respond to the ethnicity question. Participants reported their gender identity as women (*n* = 98, 71.01%), men (*n* = 44, 31.88%), non-binary (*n* = 2, 1.45%), and 15 (10.87%) did not provide a response.

### 6.2 Procedure and measures

Participation occurred in-person in a campus laboratory with paper/pencil questionnaires (*n* = 43), or online via a Qualtrics survey (*n* = 95). Instructions indicated “We are interested in your beliefs about allowing enough time to get at least 8 h of sleep each night for the next week of your life (the next 7 days).” Following the procedures of Fishbein and Ajzen (2010), participants were presented with eight open-ended questions to capture behavioral, normative, and control beliefs.

To elicit attitude-relevant behavioral beliefs, participants responded to two questions: “*What do you believe are the advantages (disadvantages) of allowing enough time to get at least 8 h of sleep each night for the next 7 days?.”* Four questions were designed to generate normative beliefs. Two were designed to capture injunctive normative beliefs: *“Please list the individuals or groups who would approve (disapprove) of you allowing enough time to get at least 8 h of sleep each night for the next 7 days (indicate their relation to you rather than their name),”* and two questions were designed to obtain descriptive normative beliefs: “*Please list the individuals who are most (least) likely to allow enough time to get at least 8 h of sleep each night.”* In addition, participants responded to two questions regarding control beliefs: *What factors or circumstances make it difficult or prevent you from allowing (easy or enable you to allow for) enough time to get at least 8 h of sleep each night for the next 7 days?*

## 7 Study 1 results

To identify the “modal set of salient or readily accessible beliefs” (Fishbein and Ajzen, 2010, p. 102), a content analysis was conducted to categorize the most frequently reported behavioral, normative, and control beliefs. Participant responses were organized by grouping together similar beliefs into categories and counting the frequencies for each category. Three individuals (the lead author plus two research assistants) read and independently categorized responses collected from the in-person participants. Results were discussed, revealing very high agreement. Given the strong consensus on the identified categories, the lead author used these categories to independently tally responses collected from the remaining (online) participants. Following a recommendation by Fishbein and Ajzen (2010), categories were retained in each set of beliefs until at least 75% of participant responses were accounted for.

### 7.1 Behavioral beliefs

The most frequently reported advantage of allowing time for 8+ hours of sleep was categorized as “Productivity.” Included were the outcomes of feeling more “productive,” “motivated,” “refreshed,” “energized,” and “less tired.” Most participants (67.36%) reported at least one of these outcomes. The next most reported advantage (37.89% of participants) was “Thinking.” Included in this category were “thinking clearly,” “better decision making,” “learning better in class,” and “better concertation.” “Emotional Benefits” were also frequently reported (32.63% of participants). Included in this category were “less moody,” “happier,” “less annoyed with others,” and “reduced stress.” Lastly, “Physical Health” was identified by 10.52% of participants which included “physical/athletic benefits,” “stronger immune system,” and “lower risk of disease.”

A frequently expected disadvantage of allowing time for 8+ hours of sleep was categorized as “Less Time” (77.89% of participants). This included expectations of having less time to “work,” “complete homework,” “get things done,” and “socialize.” This category also included participants expecting that allowing time for 8+ hours of sleep results in “schedule conflicts,” “losing free time,” “missing time with family,” and “FOMO” (fear of missing out). The only other somewhat frequently reported disadvantage was “Too Much Sleep” (29.46% of participants). Included in this category were “too much sleep makes me more tired,” “feeling sluggish,” and “feeling more stressed.”

### 7.2 Normative beliefs

The most frequent normative referents exerting injunctive pressure were “Family,” with “parents” or “siblings” nearly always mentioned. Most participants (86.20%) reported that their family would approve of them allowing time for 8+ hours of sleep, whereas only 12.63% reported that their family would disapprove. Additional referents were “Professors/Instructors/Teachers” (40.00% of participants expecting approval, 10.53% expecting disapproval), “Friends” (36.64% approval, 31.58% disapproval), “Significant Other” including boyfriend/girlfriend/spouse (21.05% approval, 4.21% disapproval), “Doctors/Nurses/Therapists/Counselors” (18.95% approval, 0% disapproval), and “Coworkers/Employers” (8.42% approve, 6.31% disapprove).

Descriptive referents exerting injunctive pressure were similar. The most frequently category was again “Family” with 46.32% of participants reporting that family members are likely to allow time for 8+ hours of sleep, and 20.00% reported that their family is unlikely to do so. Also similar were “Professors/Instructors/Teachers” (10.53% likely, 2.08% unlikely), “Friends” (5.21% likely, 21.05% unlikely), and Employed/Working Individuals (14.74% likely, 10.53% unlikely). A unique category emerged for injunctive referent: “Students” (including college/young adults/teenagers). 28.42% of participants reported that students are not likely to allow time for 8+ hours of sleep, whereas only 6.32% of participants reported they are likely.

### 7.3 Control beliefs

“Schoolwork” was the most reported factor that would make it difficult to allow time for at 8+ hours of sleep (69.47% of participants). This includes “homework,” “studying,” and “early classes.” Second most common was “Social Activities” (36.89% of participants) which includes “going out late,” “friends,” and “social events.” Somewhat frequent were responses categorized as “Technology Use” (28.42% of participants) including “using phone,” “video games,” “social media,” “movies and TV,” and “screen time.” Lastly, “Difficulty Falling or Staying Asleep,” which included reports of “anxiety,” “stress,” and “insomnia,” was mentioned by 17.89% of participants.

The most reported factor that would make it easy to allow time for at least 8 h of sleep were behaviors categorized as “Self-Care/Relaxing,” mentioned by 38.90% of participants. This category included “quiet room,” “comfortable blanket/bed/pajamas,” “no light,” “no caffeine,” and “reading a book.” Also reported was “Good Time Management” (23.16% of participants) which included “setting a schedule,” “going to bed at a decent time,” “good time management,” and “a consistent schedule.”

## 8 Study 1 summary

This elicitation study identified behavioral, normative, and control beliefs about allowing enough time to obtain 8+ hours of sleep every night. Identifying these beliefs is necessary for understanding why some individuals engage in and others do not engage in this important behavior. Specifically, identification of expected outcomes is necessary toward the goal of understanding why individuals hold positive or negative attitudes toward the behavior. Similarly, identification of influential people is necessary toward understanding why individuals perceive normative pressure to engage (or not engage) in the behavior. Likewise, identification of control beliefs is necessary to understand why individuals report high or low levels of PBC. Toward these goals, the next necessary step was to conduct a validation study to empirically test which of the beliefs identified in Study 1 are most important. To do so, belief-based expectancy value measures were created for Study 2, and used to predict direct measures of attitude, PNP, and PBC.

## 9 Study 2 method

### 9.1 Participants

Undergraduates (*N* = 850) at a large Midwestern USA university were recruited from an introductory psychology participant pool and received course credit for participation. Their mean age was 18.92 years (SD = 2.38). Participants reported as White/Caucasian (*n* = 598, 70.40%), Black/African American (*n* = 86, 10.10%), Hispanic (*n* = 56, 6.60%), Asian/Pacific Islander (*n* = 18, 2.10%), other/prefer to not say (*n* = 86, 10.1%), Native American (*n* = 5, 0.6%), and 1 participant did not respond to the ethnicity question. Participants reported as female (*n* = 663, 78.00%), male (*n* = 181, 21.30%), prefer to not answer (*n* = 5, 0.60%) and one participant did not respond.

### 9.2 Measures and procedure

Participants responded online via Qualtrics to questions concerning their “opinions toward allowing enough time for at least 8 h of uninterrupted sleep each night for the next week (the next 7 days).” Participants first responded to direct measures of attitude, PNP, and PBC, and then to belief-based expectancy value items, as detailed below.

#### 9.2.1 Direct measures

Participants responded on 7-point bipolar scales to the direct measures of attitude, PNP, PBC, and behavioral intention created for Study 3 of Tagler et al. (2017). These items were presented in randomized order.

##### 9.2.1.1 Attitude

Participants evaluated “For me, allowing enough time for at least 8 h of sleep each night for the next week is:” on the following scales assessing both experiential and instrumental components of attitude: good–bad, positive–negative, valuable–worthless, unpleasant–pleasant, nice–awful, enjoyable–unenjoyable, harmful–beneficial, wonderful–awful, boring–appealing, necessary–unnecessary, foolish–wise, and detrimental–constructive. Responses to the 12 items were averaged to produce a reliable measure (Cronbach’s *α* = 0.93) with higher scores indicating more positive attitudes. On average, participants reported favorable attitudes (*M* = 5.84, *Md* = 6.08, SD = 1.00).

##### 9.2.1.2 Perceived normative pressure (PNP)

Participants responded to nine items comprising both injunctive (e.g., “Most people who are important to me think that I should allow time for 8 h of sleep each night”; 1 = definitely true, 7 = definitely false) and descriptive (e.g., “Most people I respect and admire allow time for 8 h of sleep each night”; 1 = unlikely, 7 = likely) normative pressure. Responses were averaged to produce a reliable measure (*α* = 0.75) with higher scores indicating greater PNP. Participants perceived moderate pressure to allow time for 8+ hours of sleep (*M* = 4.80, *Md* = 4.89, SD = 0.92).

##### 9.2.1.3 Perceived behavioral control (PBC)

Participants responded to eight items including both capacity and autonomy aspects of PBC. Items include “For me to allow time for 8 h of sleep each night for the next week would be:” (1 = impossible, 7 = possible) and “It is mostly up to me whether or not I allow for 8 h of sleep each night for the next week” (1 = strongly agree, 7 = strongly disagree). Responses were averaged to produce a reliable measure with higher scores indicating greater PBC (*α* = 0.90). Average PBC was slightly above the neutral point of the scale (*M* = 4.70, *Md* = 4.75, SD = 1.31).

##### 9.2.1.4 Behavioral intention

Participants answered seven intention questions, including: “I intend to allow enough time for 8 h of sleep each night for the next week” (1 = definitely true, 7 = definitely false). Responses were averaged to create a total with higher scores indicating greater intention (*α* = 0.91). On average there was moderately favorable intention (*M* = 4.83, *Md* = 4.86, SD = 1.39).

#### 9.2.2 Belief-based items

The beliefs identified in the elicitation study (Study 1) were used to create 26 pairs of questions for the purpose of computing expectancy-value products (Fishbein and Ajzen, 2010). Pairs were presented in a randomized order to each participant.

##### 9.2.2.1 Behavioral beliefs

Nine expected outcomes (advantages/disadvantages) identified in Study 1 were used to create items (see Table 1) measuring Belief Strength (B) and Outcome Evaluation (E) on 7-point bi-polar scales. For example, participants responded to “Allowing myself time for 8 h of sleep each night for the next week will result in having more energy” (−3 = extremely unlikely, +3 = extremely likely), followed by “For me, having more energy is:” (−3 = Bad, +3 = Good).

**Table 1:** Multiple regression predicting intentions to allow time for 8 h of sleep (*N* = 850).

	*b*	SE	*β*
Attitude	0.45	0.04	0.32
Perceived normative pressure	0.40	0.04	0.26
Perceived behavioral control	0.42	0.03	0.39

*b*, unstandardized coefficient; SE, standard error of the unstandardized coefficient; *β*, standardized coefficient; all coefficients *p* < 0.001.

##### 9.2.2.2 Normative beliefs

Six injunctive normative referents identified in Study 1 were used to create items (see Table 2) measuring Strength of Normative Belief (N) and Motivation to Comply (M) on 7-point response scales. For example, “My family thinks that ____ allow time for 8 h of sleep each night.” (−3 = I should, +3 = I should not; reverse scored) followed by “When it comes to my sleep habits, I want to do what my family thinks I should do.” (−3 = agree, +3 = disagree; reverse scored).

**Table 2:** Outcome belief strength, outcome evaluation, belief-evaluation product, and correlation with direct measure of attitude (*N* = 850).

Behavioral beliefs	Outcome belief strength (B)		Outcome evaluation (E)		B × E		B × E – attitude Pearson *r* correlation
	*M*	SD	*M*	SD	*M*	SD	
Improved mood	1.92	1.47	2.62	0.93	5.60	4.24	0.48
Mental focus and concentration	2.05	1.35	2.72	0.74	5.97	3.98	0.46
Better physical health	2.10	1.27	2.68	0.79	6.04	3.83	0.44
Having more energy	1.99	1.41	2.60	0.89	5.66	4.13	0.42
More productivity	1.67	1.66	2.67	0.85	4.83	4.86	0.42
More stressed	−1.20	1.89	−2.62	0.97	3.66	5.39	0.38
Will feel more tired	−1.05	1.86	−2.43	1.05	3.03	5.17	0.38
No time to get everything done	0.16	2.06	−2.38	1.31	−0.09	5.87	0.25
Missing out on social activities	−0.36	2.12	−1.06	1.54	0.04	4.40	0.16

All correlations are significant at *p* < 0.001.

In addition, five descriptive normative referents identified in Study 1 were used to create items (see Table 2) measuring Strength of Normative Belief (N) and Identification with Referent (I) on 7-point response scales. For example, “Professors/Instructors typically allow themselves time for 8 h of sleep each night.” (−3 = false, +3 = true), followed by “When it comes to sleep habits, how much do you want to be like Professors/Instructors?” (−3 = not at all, +3 = very much).

##### 9.2.2.3 Control beliefs

Six control beliefs identified in Study 1 were used to create items (see Table 3) measuring Control Factor Strength (C) and Power of Control Factor (P) on 7-point response scales. For example, “I will have good time management for the next week.” (−3 = likely, +3 = unlikely; reverse scored), followed by “Good time management would enable me to allow for 8 h of sleep each night.” (−3 = disagree, +3 = agree).

**Table 3:** Normative belief strength, motivation to comply, normative-motivation product, and correlation with direct measure of normative pressure (*N* = 850).

Injunctive referents	Normative belief strength (N)		Motivation to comply (M)		N × M		N × M – PNP Pearson *r* correlation
	*M*	SD	*M*	SD	*M*	SD	
Family	2.41	1.01	1.45	1.64	4.12	4.65	0.37
Doctor/therapist	2.67	0.83	1.99	1.42	5.79	4.19	0.34
Significant other	1.71	1.43	1.13	1.71	3.36	4.40	0.32
Professors/instructors	2.16	1.23	1.08	1.75	3.21	4.69	0.30
Friends	1.55	1.45	0.73	1.83	2.60	4.19	0.30
Boss/coworkers	1.63	1.41	0.59	1.84	2.17	4.53	0.27

Descriptive normative referents	Normative belief strength (N)		Identification with referent (I)		N × I		N × I – PNP Pearson *r* correlation
	*M*	SD	*M*	SD	*M*	SD	
Family	0.46	1.94	0.36	1.76	2.59	3.60	0.13
Professors/instructors	0.66	1.53	0.33	1.53	1.62	3.13	0.14
Friends	−0.89	1.82	−0.67	1.68	2.60	3.62	0.01^ns^
Employed individuals	−0.17	1.75	−0.07	1.68	2.07	3.12	0.05^ns^
College students	−2.00	1.32	−1.38	1.53	3.71	4.00	0.03^ns^

All correlations are significant at *p* < 0.001 except those marked^ns^.

## 10 Study 2 results

### 10.1 Direct measures test of the RAA

Prior to engaging in the primary analyses, the RAA was tested by examining the degree to which attitude, PNP, and PBC predict intention to allow time for at least 8 h of sleep each night. The multiple regression model significantly predicted intention, *F*(3, 846) = 466.31, *p* < 0.001, and accounted for a large proportion of variability (*R*^2^ = 0.62). The analysis did not reveal a multicollinearity problem (tolerance values 0.67–0.72, VIF values 1.38–1.50). Similar to the Tagler et al. (2017) results, all predictors were statistically significant (*p*s < 0.001) and the standardized coefficients indicate that PBC was the strongest predictor (see Table 1).

### 10.2 Belief validation

Following the expectancy-value procedures of Fishbein and Ajzen (2010), the following multiplicative products were computed for each participant: B × E [the multiplication of Outcome Belief Strength (B) and Outcome Evaluation (E)], N × M [Normative Belief Strength × Motivation to Comply (M)], N × I [Normative Belief Strength (N) × Identification with Referent (I)], and C × P [Control Belief Strength (C) × Power of Control Factor (P)]. The strength of the relationship (Pearson *r*) was computed between each expectancy-value product and its corresponding direct measure of attitude, PNP, or PBC.

Table 2 presents the results for behavioral beliefs. Each B × E product significantly correlated to attitude (*p* < 0.001). Interestingly, beliefs regarding the advantages of allowing time for 8+ hours sleep (e.g., improved mood, more productivity) were both more strongly held and more predictive of attitude than the beliefs regarding disadvantages (e.g., feeling more stressed/tired, missing out).

The top of Table 3 presents the results for the injunctive normative referents and the bottom presents the results for the descriptive normative referents. Regarding the injunctive referents, each of the N × M products significantly correlated with PNP (*p* < 0.001). Belief strength and correlation with PNP were strongest for Family, followed by Doctor, Significant Other, and Professors/Instructors. Interestingly, belief strength and motivation to comply were somewhat lower for Friends and coworkers.

Regarding the descriptive referents, the belief strengths and overall results were much weaker. Only the N × I products regarding Family and Professors/Instructors were statistically significant predictors of PNP, but these correlations were small in magnitude. Beliefs about Friends, College Students, and Employed Individuals were not related to PNP. It is notable that Family/Doctor/Professors elicited the highest motivation to comply. On the other hand, there is room to increase motivation to comply with family and significant other.

Table 4 presents the results for the control beliefs. Each C × P product was significantly correlated with PBC (*p* < 0.01), with the strongest corresponding to Good Time Management (positively associated with PBC) and Having a Lot of Schoolwork (negatively associated with PBC). Further, power of control was greatest for these two beliefs, indicating that participants believe these are influential factors of why they are able or not able to successfully allow time for 8+ hours of sleep. Participants reported a high expectation (belief strength) that they would have a lot of schoolwork in the next week, but a weaker expectation that they would have good time management.

**Table 4:** Control belief strength, power of control, belief-evaluation product, and correlation of with direct measure of attitude (*N* = 850).

Control belief	Control belief strength (C)		Power of control (P)		C × P		C × P – PBC Pearson *r* correlation
	*M*	SD	*M*	SD	*M*	SD	
Good time management	0.86	1.79	1.76	1.54	1.79	4.72	0.45**
A lot of schoolwork	1.99	1.44	1.54	1.80	3.95	4.82	−0.40**
Difficulty falling/staying asleep	0.67	2.12	1.42	1.87	2.87	5.09	−0.24**
Taking time for self-care/to relax	1.06	1.83	1.40	1.74	2.15	4.78	0.22**
Using technology	1.73	1.58	1.17	1.88	2.63	4.92	−0.11*
Many social activities	0.78	1.94	0.28	2.04	1.34	4.59	−0.09*

**p < 0.001; *p < 0.01.

## 11 General discussion

The present paper advanced RAA (Fishbein and Ajzen, 2010) research on sleep behaviors. A belief elicitation study with college students identified the salient behavioral, normative, and control beliefs regarding allowing enough time for 8+ hours of sleep each night. A validation study was then conducted to identify the most important beliefs. Identification of the important beliefs is critical toward the development of effective interventions to improve sleep.

In the elicitation study, the most readily accessible (i.e., commonly reported) and important behavioral beliefs (i.e., predictive of attitude) were that allowing time for 8+ hours of sleep leads to improved mood, mental focus/concentration, physical health, and energy/productivity. In the examination of normative influence, a large majority of participants perceive that their family approves of allowing time for 8+ hours of sleep. Furthermore, this influence from family was the strongest predictor of PNP. Less frequently reported referents were doctor/therapist, significant other, professors/instructors, friends, and coworkers, but the influence from each of these were also significantly predictive of overall PNP. There does appear to be plenty of room for increases in motivation to comply with these referents. Perhaps surprising, belief strength and motivation to comply with friends were relatively low. Important control beliefs concerned the importance of good time management and schoolwork making it difficult to allow time for 8+ hours of sleep.

A simple conclusion to draw from the correlational results is that interventions to improve sleep duration should be designed in which participants: 1. Are persuaded to endorse the psychological and health benefits of healthy sleep duration, 2. Increase their awareness of expectations from family and other normative referents to get enough sleep, and 3. Receive time management training. However, the descriptive statistics of the expectancy-value measures provide additional important information that should lead to better focused interventions.

Specifically, the mean belief strength and outcome evaluation values in Table 2 indicate that many participants already hold favorable beliefs about the benefits of 8+ hours of sleep and there is not much room for further persuasion. However, there was one outcome that participants somewhat less strongly endorsed: More productivity. Given this result, it may be effective to design an intervention to persuade individuals that additional sleep can lead to greater daily productivity. Regarding normative influence (Table 3), participants strongly believe that family wants them to allow time for sleep. But there is relatively low motivation to comply with this expectation. Thus, targeting this motivation may be an additional effective strategy to increase overall PNP.

Perhaps the most important implication from both the present and prior studies is that increasing PBC should be a major component of sleep behavior interventions. As in many earlier studies, the direct measure of PBC was the strongest predictor of intention. Moreover, there is room for significant PBC increase. PBC was most strongly predicted by the specific belief that good time management will allow for 8+ hours of sleep. The belief that schoolwork prevents time for 8+ hours of sleep was also strongly related to PBC. Recently, Peltz (2024) found that endorsement of “A college student’s sleep schedule is largely out of their control” is predictive of greater self-reported sleep disturbance. Taken together these findings indicate that helping college students improve their time management skills and increasing their perceived control may be an effective approach for sleep interventions.

A limitation of the present studies is that they used college student samples from just one university, restricting the findings’ applicability to other populations.^1^ As such the results are specific to those studied, young adults from a large Midwestern USA university. Replications and extensions are needed. Researchers examining different populations should conduct similar formative research (elicitation plus validation studies) and consider the behaviors of importance for their population. In the RAA, behaviors are defined by target, action, context, and time (Fishbein and Ajzen, 2010). Changing any element, such as altering the target to middle-aged adults, is likely to result in different patterns of results. In the present study, “allowing time for at least 8 h of sleep each night” was specifically chosen because traditionally-aged college students are still in or just beyond their teenage years, with higher sleep needs (8–10 h per night, Carskadon et al., 2004; Hirshkowitz et al., 2015) than the adult need of 7–9 h.

Because it is perhaps the most critical/fundamental aspect of sleep hygiene, the present studies focused on allowing time for adequate sleep duration. With a similar focus, both Tagler et al. (2017, Study 3) and Mead and Irish (2022) found PBC to be the strongest predictor of intention. However, differences emerged in the prediction of behavior. Tagler et al. found intention (but not PBC) to predict subsequent 7-days of actigraphy-recorded sleep duration. However, Mead and Irish reported a more complicated pattern of results. First, although the within-persons effect of intention predicted sleep opportunity (i.e., individual’s earlier stated intentions predict actual sleep opportunity later that same day) the between-persons effect was not significant (i.e., whether an individual’s intention was higher/lower relative to the intentions of other participants). Moreover, intentions to allow time to sleep 8–9 h significantly decrease over the course of an individual day. Thus, for many individuals it appears that intentions to get a full night of sleep fade later in the day, and it is these later intentions that are a better predictor of actual sleep timings. This finding is important and is an example of the intention – behavior gap problem (Sheeran and Webb, 2016). From a pure prediction accuracy perspective, it is better to measure sleep intentions later in the day (when intention is more proximal to behavior). But from an applied perspective, there is a need to better understand why sleep intentions change and what can be done to strengthen/stabilize intentions so that participants are more likely to obtain sufficient sleep (Conner and Norman, 2022).

Additional EMA studies can be conducted to understand why and how intentions change. Combining the methods of the present studies with that of Mead and Irish (2022), it would be interesting to examine the stability of behavioral, normative, and control beliefs that participants report throughout the day. Might participants report different beliefs, weaker beliefs, or would the expectancy-value products of beliefs differ later in the day? Moreover, are there changes in the strength of correlations between expectancy-value products and measures of attitude, PN, and PBC throughout the day? An understanding of such changes might reveal important information for intervention efforts.

Another limitation of the present research was the absence of a behavioral measure (e.g., sleep duration, sleep opportunity assessed with actigraphy). All measures in the present studies were self-report, and as such may be influenced social desirability bias or inaccurate recall of beliefs. Moreover, a behavioral measure would allow comparisons of the beliefs and belief strengths of those who do and do not engage in the behavior (e.g., Ajzen and Driver, 1991). Thus, the comparison needed is between those who do habitually allow time for 8+ hours sleep, with those who usually do not. Doing so can provide more information for designing targeted/tailored interventions. For example, it is possible that short duration sleepers evaluate their time management ability much lower than longer duration sleepers do.

The design and testing of interventions represent the full application of the RAA: Moving from predicting, to understanding, to improving behavior. The present studies advanced research toward understanding why many individuals do not allow time to obtain adequate sleep. The belief elicitation and validation studies identified the salient and important beliefs that college students hold about allowing time for 8+ hours of sleep every night. With the goal of designing effective interventions to improve sleep, researchers need to continue to conduct studies to fully explain the roles of attitudes, PNP, PBC, and intention on sleep behaviors.

## Data availability statement

The raw data supporting the conclusions of this article will be made available by the authors, without undue reservation.

## Ethics statement

The studies involving humans were approved by the Ball State University Institutional Review Board. The studies were conducted in accordance with the local legislation and institutional requirements. The Ethics Committee/Institutional Review Board waived the requirement of written informed consent for participation from the participants because data were collected online and anonymously.

## Author contributions

MT: Writing – review & editing, Writing – original draft, Methodology, Formal analysis, Data curation, Conceptualization.

## Footnotes

^1^Exploratory analysis revealed few and small demographic differences. Age was not correlated (*r* < 0.09) with the direct measures or the expectancy-value belief measures. White participants and participants of color did not score significantly different on the direct measures, but female participants reported slightly more favorable attitudes than males (*d* = 0.25). Participants of color reported slightly greater behavioral belief in the outcome of “missing out on social activities” (*d* = 0.15) and females reported stronger control belief in “a lot of schoolwork” (*d* = 0.36). There were no significant sex differences in the strength of the correlations displayed in Tables 2–4, and only one race difference: the correlation between the behavioral belief “mental focus and concentration” and the direct measure of attitude was stronger for participants of color (*r* = 0.61) than for white participants (*r* = 0.41).
